# Supplementary figures and images for: Dissection of Besnoitia besnoiti intermediate host life cycle stages: From morphology to gene expression
Source: PLoS Pathog. 2022 Nov 17;18(11):e1010955. doi: 10.1371/journal.ppat.1010955 (PMC9714946; doi:10.1371/journal.ppat.1010955)

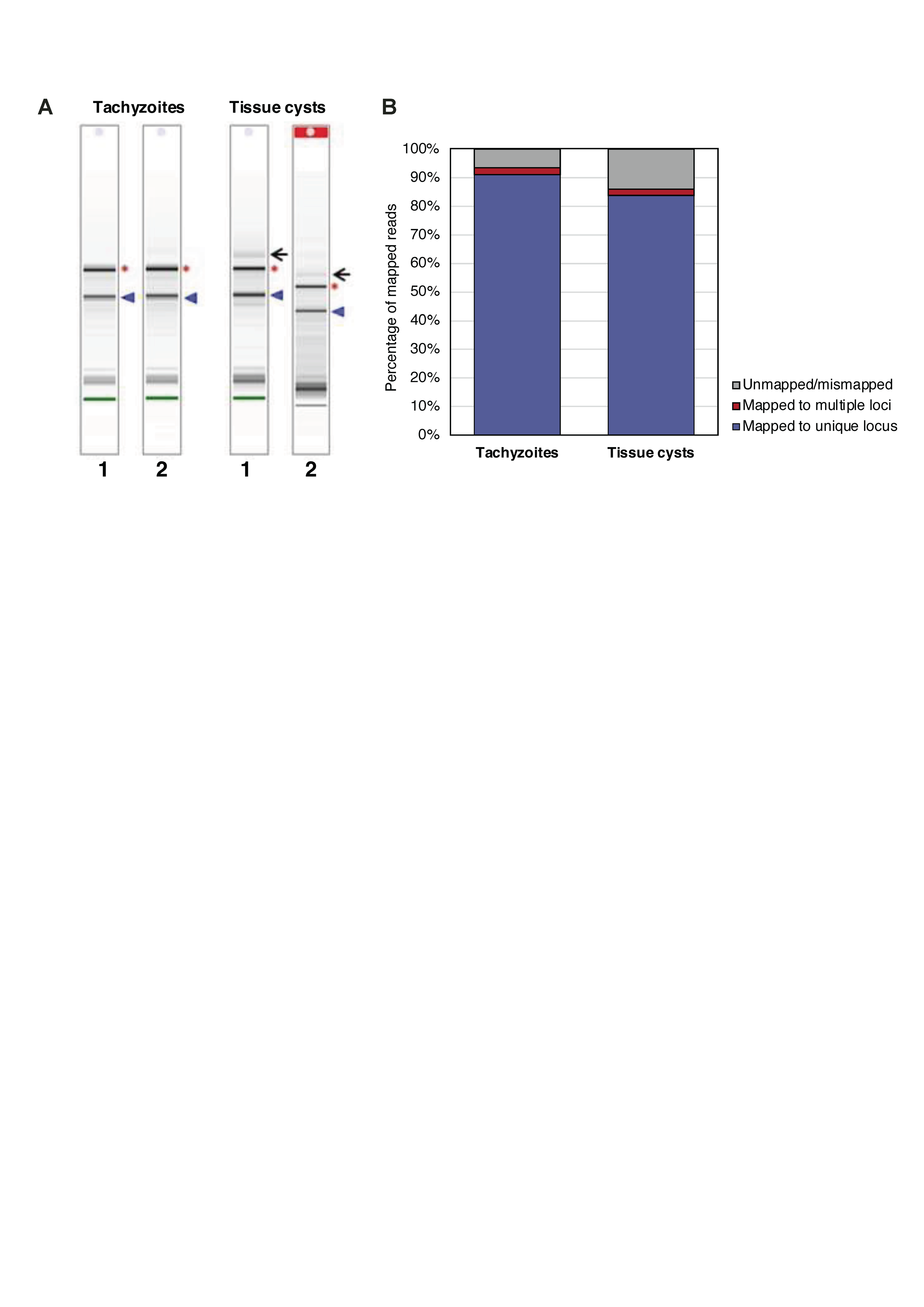

Supplement: S1 Fig — (A) Simulated gel images of RNA profiles from Bioanalyzer analyses. Shown are the two samples pooled for RNA-Seq. Red asterisks mark the parasite-specific 26S ribosomal RNA bands, arrows the host-specific 28S rRNA bands and blue arrow heads the parasite- and host-specific 18S rRNA bands. (B) Bar graph showing percentage of the unique and non-unique reads mapped to the B. besnoiti genome. (TIF) [file ppat.1010955.s001.tif]

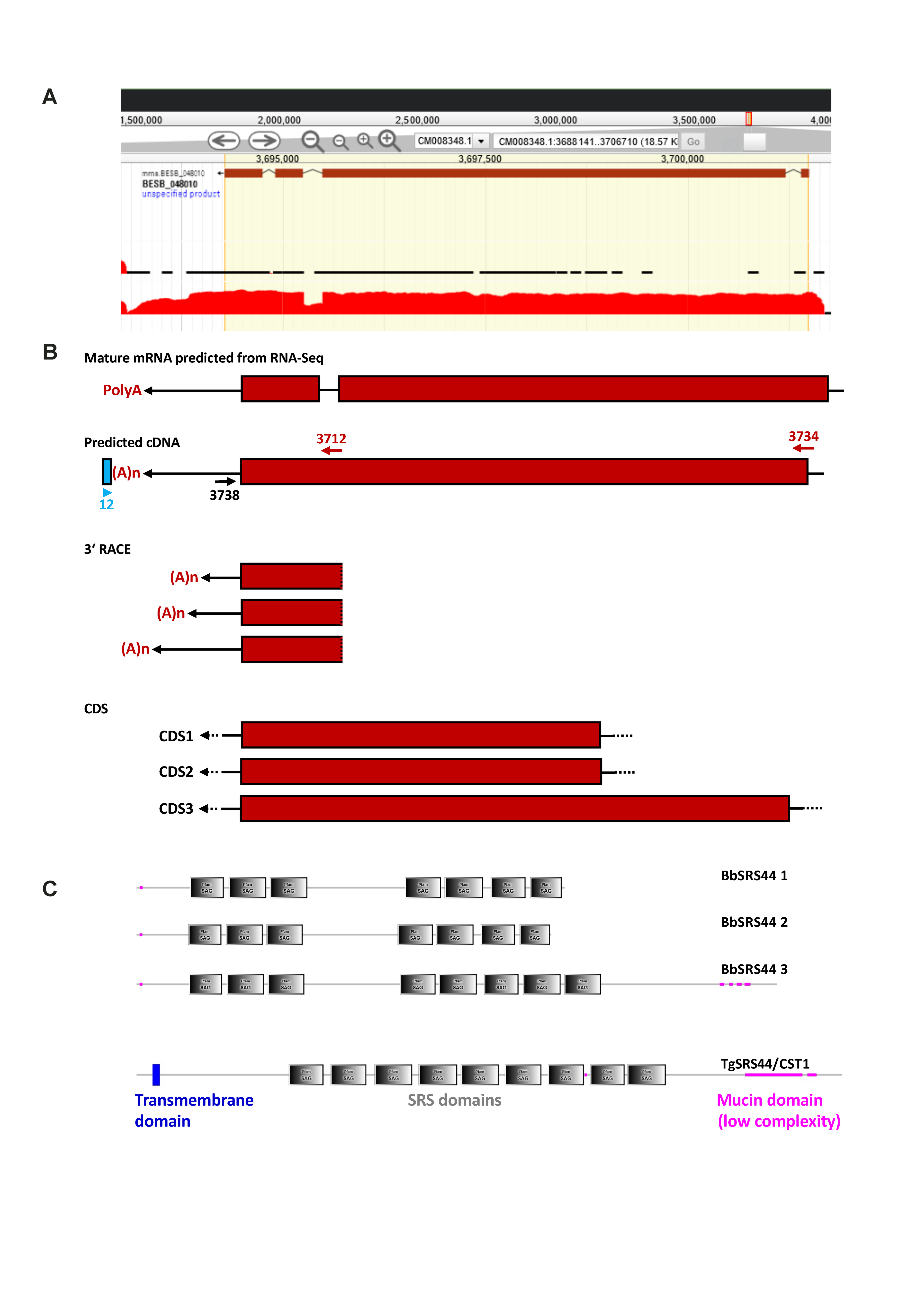

Supplement: S2 Fig — (A) Gene model and mapped RNA-Seq reads (ToxoDB release 53). (B) Predicted transcripts of B. besnoiti SRS44, model inferred using RNA-Seq data and a model for 3’ end resulting from RACE PCR data and CDS from primer walking. (C) Domain structures of B. besnoiti and T. gondii SRS44. (TIF) [file ppat.1010955.s002.tif]

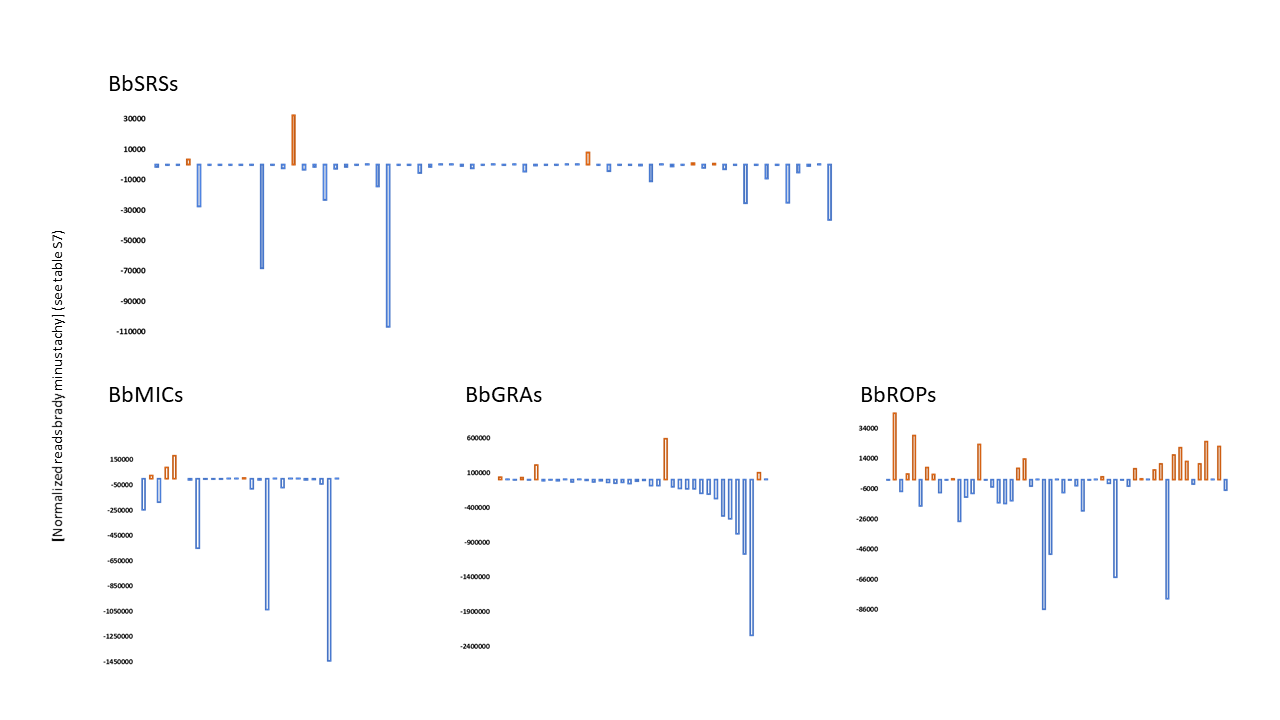

Supplement: S3 Fig — Genes overexpressed in bradyzoites or in tachyzoites are indicated in orange and blue, respectively. (TIF) [file ppat.1010955.s003.tif]

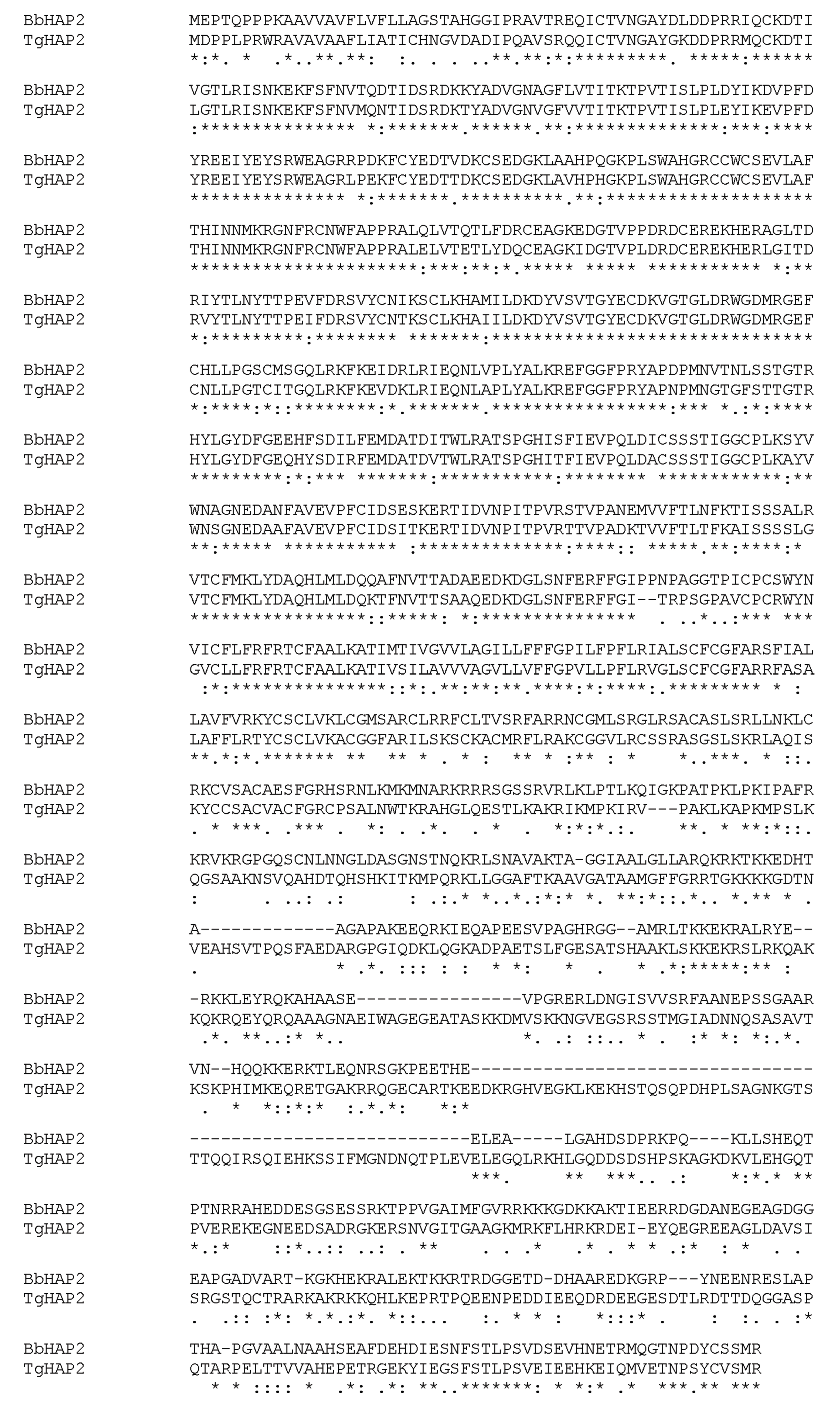

Supplement: S4 Fig — Asterisks mark identical, colons very similar, and dots similar amino acids. (TIF) [file ppat.1010955.s004.tif]

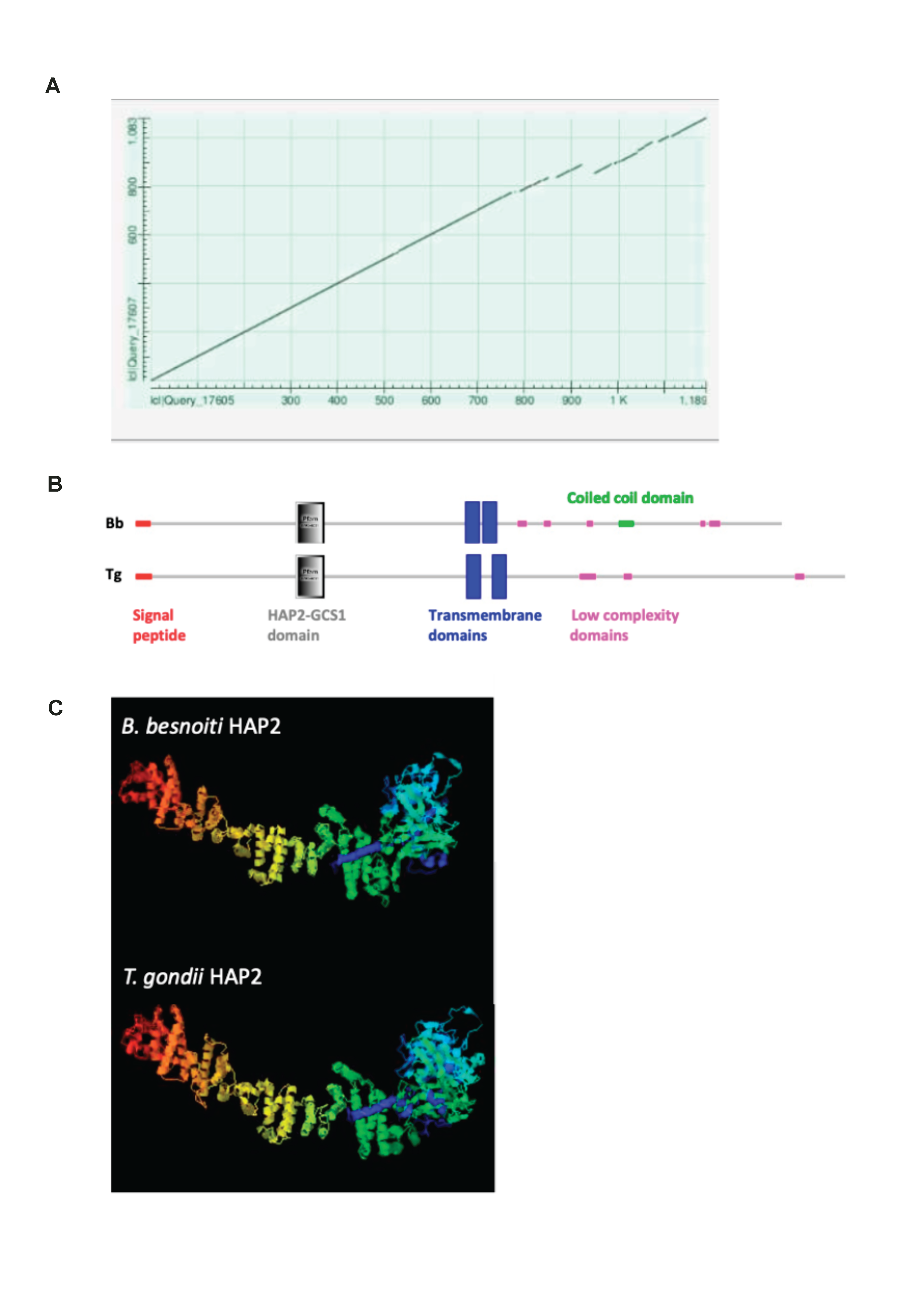

Supplement: S5 Fig — (A) Dotplot of the sequence alignment. (B) Domain predictions of B. besnoiti and T. gondii HAP2 using SMART. (C) Prediction of 3D structure of B. besnoiti and T. gondii HAP2 using I-TASSER. (TIF) [file ppat.1010955.s005.tif]
