# Supplementary material for: Dissection of Besnoitia besnoiti intermediate host life cycle stages: From morphology to gene expression
Source: PLoS Pathog. 2022 Nov 17;18(11):e1010955. doi: 10.1371/journal.ppat.1010955 (PMC9714946; doi:10.1371/journal.ppat.1010955)
Supplement: Attachment — Submitted filename: Response_PPATHOGENS-D-22-01043.docx [file ppat.1010955.s017.docx]

Editors

PLoS Pathogens

Zürich, September 30, 2022

Dear Editors,

Thank you again for your decision to consider a revised version of our submission PPATHOGENS-D-22-01043. We also thank the three expert Reviewers for their time to evaluate this manuscript and for their constructive suggestions and criticisms which we addressed in detail below.

We have reorganized the manuscript and adjusted the focus on data analysis comparing tachyzoite and cyst stages of *B. besnoiti*. In addition to implementing the Reviewer’s comments (minor issues), we specifically:

- Made the narrative more coherent by reorganizing the manuscript to place the analysis of sexual stage genes at the end.
- Verified that *B. besnoiti* cysts walls do not react with the *Dolichos biflorus* lectin consistent with the absence of a mucin domain on the *B. besnoiti* homolog of CST1 (see main text).
- Complemented the analysis of sexual cycle-specific genes by considering also orthologues of cyst-specific genes (see Fig. 10).

In addition, we have supplemented and reorganized the supplemental figures and data by creating or extending tables and creating new figures as suggested by the reviewers. All changes are documented in detail in our rebuttal below.

We thank you for considering this revised manuscript for publication in PLoS Pathogens, and we look forward to your response.

Sincerely,

Adrian B. Hehl, Ph.D	Chandra Ramakrishnan, Ph.D

**Reviewer's Responses to Questions**

Dear Drs A. Hehl and C. Ramakrishnan,

Thank you very much for submitting your manuscript "Dissection of Besnoitia besnoiti intermediate host life cycle stages: from morphology to gene expression" for consideration at PLOS Pathogens. As with all papers reviewed by the journal, your manuscript was reviewed by members of the editorial board and by 3 independent reviewers.

As part of the lifecycle of Besnoitia besnoiti remains puzzling, the editors and reviewers have appreciated your study representing a useful addition to the field. However, we found that the presentation of the data needs rearrangement and more accuracy in the gene names; the main purpose/message of the work is confusing; and the work will benefit from a better integration of the results with sexual stages of other Apicomplexa.

Please prepare and submit your revised manuscript within 30 days. If you anticipate any delay, please let us know the expected resubmission date by replying to this email.

When you are ready to resubmit, please upload the following:

[1] A letter containing a detailed list of your responses to all review comments, and a description of the changes you have made in the manuscript.

Please note while forming your response, if your article is accepted, you may have the opportunity to make the peer review history publicly available. The record will include editor decision letters (with reviews) and your responses to reviewer comments. If eligible, we will contact you to opt in or out

[2] Two versions of the revised manuscript: one with either highlights or tracked changes denoting where the text has been changed; the other a clean version (uploaded as the manuscript file).

Important additional instructions are given below your reviewer comments

**Part I - Summary**
Please use this section to discuss strengths/weaknesses of study, novelty/significance, general execution and scholarship.

Reviewer #1: A review of genome and RNAseq data on Besnoitia besnoiti isolated from cattle. The analysis of the sequence data is reasonable and is correlated with data in T. gondii and other Apicomplexa.

The data indicate that a sexual cycle exists and, to an extent, exclude an insect host (based on looking at Plasmodium homologs that might indicate an insect host). I do not think this is completely excluded by the data, but it is a reasonable suggestion. The light and EM images of the cysts are useful and add to the literature. In fact, a more detailed series of images in the supplemental figures could be useful.

*>We appreciate this Reviewer’s positive general comments. The suggestion has been followed (see also below).*

Reviewer #2: 1. Here the authors use microscopy, genome sequencing, and transcriptomics to characterize Besnoitia besnoiti in its in vitro tachyzoite and in vivo bradyzoite forms. Genomic comparisons to Toxoplasma gondii, Neospora caninum, Eimeria tenella, and Plasmodium falciparum are made as well as transcriptomic comparisons to T. gondii. In silico evidence for a definitive B. besnoiti host and different metabolic pathways are presented. Overall, a useful addition to the field.

- *We thank the Reviewer for this statement.*

Some confusion about the purpose of the paper- is the main idea comparison of intermediate stages as title suggests, or is it more about presenting an argument for a sexual stage and definitive host? To strengthen definitive host argument, deeper analysis of intermediate stage orthologs between T. gondii and B. besnoiti should be performed. Figure confusion needs to be addressed. Recommend some rearrangement and condensation of figures.

- *We have addressed these issues (for details see below).*

Reviewer #3: Besnoitia besnoiti is a cyst-forming apicomplexan protozoan parasite closely related to Toxoplasma gondii that can infect livestock, especially cattle, as intermediate hosts. Bovine besnoitiosis is a disease that progresses in two phases: an acute phase and a chronic phase. The acute phase can be fatal or cause infertility or sterility in males. Symptoms range from fever and anorexia to vascular disorders. In the chronic phase, various skin lesions may occur. Unfortunately, there are no vaccines or therapeutics to date that could halt the progression of the disease. This study represents a milestone in the study of Besnoitia besnoiti biology and provides a wealth of "omics" data on two different stages of the intermediate host cattle. Transmission electron microscope (TEM) analysis revealed that organellar structures match well with those of T. gondii, but also remarkable differences in the cyst wall, which has probably adapted to its particular niche in the skin.

- *We thank the Reviewer for these comments.*

A significant part of the life cycle of this parasite remains unknown, and to date no definitive host has been assigned to it. This study provides unequivocal genomic evidence for the presence of genes in Besnoitia besnoiti that are homologous to those of Toxoplasma and known to be specific for the sexual cycle (development of microgametes and macrogametes) in the cat intestine and oocyst formation, supporting the original hypothesis of a definitive carnivorous/scavenger host for this parasite. Regarding the expression of its genome, B. besnoiti has conserved a fingerprint of conserved stage-specific markers, but is also characterized by a specific subtranscriptome. As in Toxoplasma, the differences between the tachyzoite and bradyzoite stages show very distinct signatures.
The entire life cycle of B. besnoiti remains to be elucidated, but this study provides a fairly comprehensive analysis of the biology of zoites responsible for acute and chronic disease.

**Part II – Major Issues: Key Experiments Required for Acceptance**
Please use this section to detail the key new experiments or modifications of existing experiments that should be absolutely required to validate study conclusions.

Generally, there should be no more than 3 such required experiments or major modifications for a "Major Revision" recommendation. If more than 3 experiments are necessary to validate the study conclusions, then you are encouraged to recommend "Reject".

Reviewer #1: The data is clearly presented and appears complete. I do not see much need for additional validation of the RNAseq data using immunoblot or proteomic approaches; as one would expect a reasonably close correlation between the RNAseq data and results on protein levels for the genes discussed.

The author notes that the CST1 homolog in Besnoitia lacks a mucin domain and would not be a source of lectin staining; however, I did not see any mention of lectin staining of these cysts. It would be useful to provide this data (e.g show that cysts stain with Dolichus). If they are DBA positive then the issue is if this is still due to glycosylation of the CST1 homolog or if another gene exists with a mucin or similar domain that would be DBA positive if post transitionally modified.

- *We appreciate the comment and we have addressed this by providing new experimental procedures (DBA lectin binding assay) and information, and by amending the main text correspondingly. In brief, in contrast with a robust staining pattern delineating the cyst wall in differentiated T. gondii, no signal with conjugated DBA could be produced to date on B. besnoiti tissue cysts.*

Reviewer #2: Regarding in silico evidence for a definitive host: authors rely heavily on existence/number/conservation of B. besnoiti orthologs of Toxo sex genes. To strengthen this argument, why not perform a similar analysis on bradyzoite/cyst genes? Because intermediate lifestage data are available and reliable in both Toxo and B. besnoiti, that analysis should serve as a positive control for what you might expect to observe for sex genes. For example, of the B. besnoiti genes that are highly expressed in Bb cysts relative to tachyzoites, how many are orthologous to Toxo bradyzoite/cyst genes?

- *We appreciate the idea behind this suggestion. We have performed this analysis and added the results as an additional panel to Figure 10 (B) with a column graph depicting the percentage of B. besnoiti orthologues of T. gondii bradyzoite/cyst genes. However, we also caution that this comparison is not straightforward and possibly flawed because 1) the biological differences between T. gondii and B. besnoiti cysts are massive in terms of size, wall structure, and tissue localization and 2) the background of bradyzoite gene expression in B. besnoiti tachyzoites is essentially zero because the Lis14 strain is unresponsive to in vitro stress conditions and no spontaneous cyst formation has been observed in vitro. A priori, the latter results in a much lower signal-to-noise ratio in a comparative analysis of mRNA expression and therefore a higher number of significantly stage-regulated genes in B. besnoiti.*

Reviewer #3: (No Response)

**Part III – Minor Issues: Editorial and Data Presentation Modifications**
Please use this section for editorial suggestions as well as relatively minor modifications of existing data that would enhance clarity.

Reviewer #1: I would suggest that Figure S1 on the TEM of the cyst be included in the figures in the paper rather than supplemental data. In addition, if more images exist of the TEM and SEM they could be provided as additional supplemental images.

In Figure 1 higher magnification images of the light microscopy with HE and MB/fuchsin should be provided.

- *We appreciate the Reviewer’s feedback. We have now incorporated Figure S1 into Figure 2. The light microscopy images were taken on site while isolating the tissue cysts. At this time, we are unable to provide higher magnification images.*

Reviewer #2: Figure legends:
o please specify that images are of the cyst wall surface in Fig. 1D-G

- *Specification that these are images of the tissue cyst wall surface is now added to the figure legend.*

7D description is repeated. difference between 7C and D is unclear in legend, figure, and text (more below)

- *We thank the reviewer for noting this error in labeling. Previous figure 7C refers to B. besnoiti orthologues of tissue cyst-specific genes in T. gondii (Fig. 7D). The figure legend has been corrected accordingly.*

The authors write as though the most interesting part of the paper is evidence for an as-yet unknown definitive host, which is interesting. For example, in the intro and results, evidence for a definitive host is discussed and presented first. Recommend discussing/presenting sexual stage last after discussing intermediate stages.

- *We appreciate this comment and we have rearranged the manuscript chapters accordingly.*

Page 9: authors note that 432 genes were expressed in neither tachys nor bradys. how does that fraction compare to T. gondii gene expression studies? ie what fraction of T. gondii genes are not expressed in tachys or bradys?

- *This is indeed an interesting question. In B. besnoiti, 5.1% of the protein-coding genes are not expressed either in tachyzoites or bradyzoites: in contrast, a larger fraction, i.e. 12.6%, are not significantly expressed in either of the T. gondii developmental stages. Although we would not venture an interpretation of this difference given the vastly superior annotation of the T. gondii Me49 reference genome, we have nevertheless added this information to the main text (Line 194).*

Page 10/figure 4a: recommend adding an intermediate color (or multiple) between blue and red. it is difficult to distinguish the shades of red and blue, blue in particular.

- *We thank the reviewer for this suggestion. The color scheme of all figures has been revised to improve legibility.*

Page 12/figure 6c: there is no figure 6c

- *We thank the reviewer for pointing this out. We have now labelled the figure correctly.*

Page 12/figure 6: text doesn’t match figure. figure shows highest percentage of genes are EES1, text states highest percentage of genes are EES5 and oocyst

- *We thank the reviewer for pointing out these labeling errors. In the revised version this is now figure 10. The color codes have been changed to match our results. In addition, 10B shows an additional graph summarizing analysis results of cyst/bradyzoite expression as requested by Reviewer 2 (see major issues). 10C, D are the same pie charts shown in previous Figure 6 with a new color scheme.*

Page 14/figure 7: figures 7c and 7d are confusing. why are there opposite colorations for most genes if the only different between c and d is the scale? I am interpreting “difference to common mean” as only a change in scale, so if that is not correct, the difference between c and d needs to be better explained in text and figure legend.

- *This issue has also been noted by Reviewer 1 and is now amended. Please refer to answer above.*

Page 14/figures 7 and 8: as is, recommend combining and condensing figures 7 and 8. move 7a to supplement and replace with 8a, as they are quite similar. 7b is fine but doesn’t say much: could either go to supplement or have added annotation to describe which classes of genes are high and low in tachys vs. cysts. there is plenty of room to make the colored portion of the plot narrower to make room for labels. 7c/d are very confusing, perhaps remove one? the dot plots in 8c/d are ok, but could easily be communicated as heatmaps like in 7c/d, with one heatmap for Toxo & one for B. besnoiti, side-by-side. 7c/d seem very similar to 8 c/d, just different genes. Given available data for Toxo, recommend adding a Toxo gene expression heatmap next to the 7c B. besnoiti heatmap. So in summary for 7c/d and 8c/d, make all into heatmaps, each with maps of B. besnoiti alongside maps of Toxo.

- *We thank the reviewer for this constructive criticism. We have now removed figure 7A and corrected the figure legends of Figures 7C and D as mentioned above. We have also combined figures 7 and 8 into one figure and adjusted the figure legends correspondingly. We have decided to keep the dot plot format, however, as in these plots we can easily depict the non-regulated genes as well, especially when illustrating the whole transcriptome.*

Reviewer #3: The manuscript contains a large amount of data, some of which is unfortunately inadequately presented. Some rewording is necessary to guide the reader through concepts that may be difficult for those outside the field to understand.

Below are some points to improve the clarity and presentation of the data:

1- Differential expression of SRS genes, ROP, MIC, and GRA genes (including newly discovered/renamed genes) should be represented as in Figure 3 of Hehl et al. (BMC Genomics, 2015). A similar graphical representation could also highlight conservation with Toxoplasma and other CFAs.

- *We appreciate this suggestion and have generated new tables specifically for these gene families in B. besnoiti as well as a corresponding supplemental Figure (S3) with column graphs illustrating stage-specific differences in expression (Table S7). Comprehensive information about these families and their conservation in T. gondii, N. caninum. E. tenella and P. falciparum is now provided in Table S7.*

2- The authors should better document the conservation of proteins responsible for interaction with host cells, especially the ROP and GRA proteins of B. besnoiti, by specifying their polymorphism, which has often been associated with their function (e.g. ROP16) or stage-specific expression (e.g. ROP18) or even interaction with their partner, e.g. KIM domains of GRA24 as drivers of host MAPK p38-alpha interaction and activation.

- *Although the reviewer raises interesting points, we would argue that neither the quality of the B. besnoiti gene models and orthologue calling, nor their biological validation are currently sufficient to support such deep analyses. In addition, comparative analysis of polymorphism-dependent virulence phenotypes requires corresponding strains and infection platforms which are currently unavailable for B. besnoiti. However, we have generated several new tables and figures which will help readers structure the data and can serve as guides for researchers in the field by providing entry points for future structure-function analyses.*

3- AP2 factors deserve special attention and should be presented in a scheme with their structural domain (AP2, ACDC) together with their putative Toxoplasma, but also Eimeria orthologs, indicating at which stage they are expressed in these parasites. Identification of MORC-associated AP2 orthologs would provide further evidence that epigenetic mechanisms exist in *B. besnoiti* to control sexual commitment.

- *A new analysis is presented in the supplemental data documenting orthologues of AP2s, MORC and associated factors in B. besnoiti as well as the virtually complete conservation of MORC-regulated microgamete genes (Tables S10 and S11)*.

4- To identify genes likely involved in the B. besnoiti sexual cycle, the authors looked for known homologs in Toxoplasma, but what about conservation in Eimeria? Are the 267 transcripts mentioned in the study unique to B. besnoiti or are they conserved in some CFAs?

- *We have addressed this comment by creating an extended table showing conservation of T. gondii EES-RO genes in B. besnoiti, E. tenella, N. caninum, and P. falciparum (Table S9).*

5- Table S7 should list TgIST (TGME49_240060), TgNSM (2351140), and TEEGR as bona fide proteins that are presents in dense granule.

- *For these genes (TGME49_240060, TGME49_235140, TGME49_239010) no B. besnoiti orthologs could be identified in our datasets or in ToxoDB. This information has been added to the table (Table S6 in the revised paper).*

PLOS authors have the option to publish the peer review history of their article ([what does this mean?](https://journals.plos.org/plospathogens/s/editorial-and-peer-review-process#loc-peer-review-history)). If published, this will include your full peer review and any attached files.


If you choose “no”, your identity will remain anonymous but your review may still be made public.


**Do you want your identity to be public for this peer review?** For information about this choice, including consent withdrawal, please see our [Privacy Policy](https://www.plos.org/privacy-policy).

Reviewer #1: No

Reviewer #2: **Yes:** Laura Knoll

Reviewer #3: No

Figure Files:

While revising your submission, please upload your figure files to the Preflight Analysis and Conversion Engine (PACE) digital diagnostic tool, https://pacev2.apexcovantage.com. PACE helps ensure that figures meet PLOS requirements. To use PACE, you must first register as a user. Then, login and navigate to the UPLOAD tab, where you will find detailed instructions on how to use the tool. If you encounter any issues or have any questions when using PACE, please email us at figures@plos.org.

Data Requirements:

Please note that, as a condition of publication, PLOS' data policy requires that you make available all data used to draw the conclusions outlined in your manuscript. Data must be deposited in an appropriate repository, included within the body of the manuscript, or uploaded as supporting information. This includes all numerical values that were used to generate graphs, histograms etc.. For an example see here: <http://www.plosbiology.org/article/info%3Adoi%2F10.1371%2Fjournal.pbio.1001908#s5>.

Reproducibility:

To enhance the reproducibility of your results, we recommend that you deposit your laboratory protocols in protocols.io, where a protocol can be assigned its own identifier (DOI) such that it can be cited independently in the future. Additionally, PLOS ONE offers an option to publish peer-reviewed clinical study protocols. Read more information on sharing protocols at <https://plos.org/protocols?utm_medium=editorial-email&utm_source=authorletters&utm_campaign=protocols>

References:

Please review your reference list to ensure that it is complete and correct. If you have cited papers that have been retracted, please include the rationale for doing so in the manuscript text, or remove these references and replace them with relevant current references. Any changes to the reference list should be mentioned in the rebuttal letter that accompanies your revised manuscript. If you need to cite a retracted article, indicate the article’s retracted status in the References list and also include a citation and full reference for the retraction notice.
