# Supplementary material for: Dissection of Besnoitia besnoiti intermediate host life cycle stages: From morphology to gene expression
Source: PLoS Pathog. 2022 Nov 17;18(11):e1010955. doi: 10.1371/journal.ppat.1010955 (PMC9714946; doi:10.1371/journal.ppat.1010955)
Supplement: Attachment — Submitted filename: Response_PPATHOGENS-D-22-01043R1.docx [file ppat.1010955.s018.docx]

Editors

PLoS Pathogens

Zürich, October 22, 2022

Dear Editors,

Thank you for your decision concerning our submission of a revised manuscript PPATHOGENS-D-22-01043R1. We have now dealt with the two requests made by the colleagues who reviewed the revised manuscript.

Specifically, we:

- Clarified the designations of the subcellular compartments and organelles in Fig. 3 and in the text.
- Removed the reference to the lack of reactivity of the *B. besnoiti* cyst wall with DBA lectin. Although controls have been performed for the DBA staining reaction using *T. gondii* cysts (see details in the Response to Reviewers below) we recognize that the negative result obtained with *B. besnoiti* cysts cannot be properly controlled: neither can the appropriate tissue context (bovine dermis) be replicated for *T. gondii* tissue cysts nor is it currently possible to induce *B. besnoiti* stage-conversion and generate tissue cysts *in vitro*.

We thank you for considering this revised manuscript for publication in PLoS Pathogens, and we look forward to your response.

Sincerely,

Adrian B. Hehl, Ph.D	Chandra Ramakrishnan, Ph.D

**Responses to questions and modifications to manuscript PPATHOGENS-D-22-01043R1**

Dear Drs A. Hehl and C. Ramakrishnan,

Thank you very much for submitting your manuscript "Dissection of Besnoitia besnoiti intermediate host life cycle stages: from morphology to gene expression" for consideration at PLOS Pathogens. As with all papers reviewed by the journal, your manuscript was reviewed by members of the editorial board and by several independent reviewers. The reviewers appreciated the attention to an important topic. Based on the reviews, we are likely to accept this manuscript for publication, providing that you modify the manuscript according to the review recommendations.

For the Editor:

Two rectifications:
1) The morphological identification of organelle: In Fig. 3: It is speculative to identify a vacuolar compartment serving as a Ca2+ storage and sodium transport activity (VAC) in the absence of marker for this organelle. Could sections of progeny. Replace VAC by VAC?

- *We agree with the reviewer that without a specific marker for the organelle, identification of VAC based on compartment morphology is not sufficiently supported. We therefore removed the designation (VAC) in Fig. 3 as well as all references in the text (lines 130-138). All line numbers refer to the “track changes” version of the manuscript.*

The ap for apicoplast in panel C is erroneous. An apicoplast is visible just above the nucleus based on multiple membranes and global shape.

- *The designation indicating the apicoplast in figure 3 was moved to the structure identified by the reviewer.*

The inner membrane complex beneath the plasma membrane can also be shown. The IMC is an organelle.

- *We thank the reviewer for this suggestion. An additional panel D was added to Fig. 3 showing an enlarged region designated by a white rectangle in panel C. The IMC structure is clearly identifiable in panel D and indicated with yellow arrows.*
- *Changes in the text: lines 130-138 (Results and Discussion), and 902-907 (Figure legends).*

2) Regarding a cyst wall staining with DAB: the image with Toxoplasma cysts as positive controls must be shown in the manuscript as it has been done. Not as personal communication to the reviewer.

-*We take this point. However, this control experiment merely served as a technical validation of the DBA staining method used on B. besnoiti cysts. The method per se is widely used and routine. The example below illustrates the robust signal obtained with T. gondii cysts. However, in the absence of a clear reaction of DBA with B. besnoiti tissue cysts and in the light of the widely different tissue context, we decided to remove the reference to this negative result in Results and Discussion (lines 287-291) and Materials and Methods (lines 521-526), which clearly needs more careful investigation after development of an in vitro model for encystation as explained in the cover letter. We hope the editors agree that removal of this minor point has no impact on the paper.*
